# Assessing household wealth in health studies in developing countries: a comparison of participatory wealth ranking and survey techniques from rural South Africa

**DOI:** 10.1186/1742-7622-4-4

**Published:** 2007-06-01

**Authors:** James R Hargreaves, Linda A Morison, John SS Gear, Julia C Kim, Mzamani B Makhubele, John DH Porter, Charlotte Watts, Paul M Pronyk

**Affiliations:** 1 London School of Hygiene and Tropical Medicine, Keppel Street, London WC1E 7HT, UK; 2 Rural AIDS and Development Action Research Programme, PO Box 2, Acornhoek 1360, South Africa

## Abstract

**Background:**

Accurate tools for assessing household wealth are essential for many health studies in developing countries. Household survey and participatory wealth ranking (PWR) are two approaches to generate data for this purpose.

**Methods:**

A household survey and PWR were conducted among eight villages in rural South Africa. We developed three indicators of household wealth using the data. One indicator used PWR data only, one used principal components analysis to combine data from the survey, while the final indicator used survey data combined in a manner informed by the PWR. We assessed internal consistency of the indices and assessed their level of agreement in ranking household wealth.

**Results:**

Food security, asset ownership, housing quality and employment were important indicators of household wealth. PWR, consisting of three independent rankings of 9671 households, showed a high level of internal consistency (intraclass correlation coefficient 0.81, 95% CI 0.79–0.82). Data on 1429 households were available from all three techniques. There was moderate agreement in ranking households into wealth tertiles between the two indicators based on survey data (spearman rho = 0.69, kappa = 0.43), but only limited agreement between these techniques and the PWR data (spearman rho = 0.38 and 0.31, kappa = 0.20 and 0.17).

**Conclusion:**

Both PWR and household survey can provide a rapid assessment of household wealth. Each technique had strengths and weaknesses. Reasons for differences might include data inaccuracies or limitations in the methods by which information was weighted. Alternatively, the techniques may measure different things. More research is needed to increase the validity of measures of socioeconomic position used in health studies in developing countries.

## Background

Research into the socioeconomic determinants of health requires accurate tools for assessing socioeconomic position. While in developed countries pre-existing data are often available, these resources rarely exist in developing countries and original data must be collected [1]. Economists generally regard detailed data on household income and/or expenditure as the gold-standard measure of current socioeconomic position. However, health researchers rarely have the resources or expertise necessary to conduct such assessments. Furthermore, total wealth, reflecting the balance between income and expenditure over a longer period, may be a more appropriate marker of socioeconomic position when health outcomes are considered. Consequently, rapid techniques for assessing household wealth are needed.

A variety of proxy measures of socioeconomic position have been developed. These have included shortened income or expenditure questionnaires, and measures of housing quality, education or nutritional status [1] Recently, researchers have used statistical techniques to combine multiple socioeconomic variables, usually including at least data on housing and ownership of fixed assets, into a measure of household wealth. The aggregation of such data can be achieved through a simple count, weighting of variables based on local consultation, or through the application of statistical procedures such as principal components analysis (PCA) [2-7]. However, there is no consensus on what variables should be included in such analyses [8]. Furthermore, there remains limited evidence on the association between asset indices and more established measures of wealth or socioeconomic position [9,10].

An alternative technique is to use participatory wealth ranking (PWR), in which community members rank the wealth of households in their community. This approach is widely used in development practice [11], but rarely used in health studies. PWR can generate useful statistics and provide valid information on relative wealth [12-16].

We conducted a household survey and PWR in rural South Africa. We constructed three indicators of household wealth, using the data from each of the two techniques separately and also by combining them. We assessed internal validity where this was possible, assessed agreement between the results of the techniques in their ranking of household wealth, and investigated the reasons for any differences.

## Analysis

### Methods

#### Setting

The study was conducted in eight rural villages of Limpopo Province, South Africa. The province is among the most deprived in the country, with nearly 50% of the population under 15 years old, unemployment in excess of 40%, and high levels of labour migration [17-20]. The data come from the baseline evaluations of a cluster randomised trial [21].

#### Data collection

##### Participatory Wealth Ranking (PWR)

PWR was conducted in the local language by specialised facilitators from a local development NGO (Small Enterprise Foundation, Tzaneen). Data were recorded on pre-designed data collection forms [22,23].

Community members residing in the same village section, most often women from poor households, drew a map of their residential area and listed the households on cards. Following this, groups of 4–6 residents were asked to characterise households that were "very poor", "poor, but a bit better off", and "doing OK". The proceedings of this discussion were captured by the facilitator in the form of "*general statements"*. Households were then ranked from the poorest to the wealthiest according to these definitions and piles of households of comparable wealth generated. Participants were then asked to describe the characteristics of the households in each ranking pile ("*pile statements"*). Neither the number of wealth ranks nor the number of households in each rank was determined in advance, although at least four separate piles had to be generated during the process.

The ranking process was then repeated twice more with different groups of four to six community members, so that statements were collected and each household ranked on three separate occasions.

##### Household survey

A random sample of approximately 200 dwellings from each village (total N = 1640) was visited at least three times where necessary to collect data. Interviews were conducted in the local language. Interviewers received extensive training and data entry was validated through data cleaning procedures. Questionnaires captured salient aspects of socioeconomic well-being including household members' education and employment status, details of the dwelling construction, ownership of a small number of assets, details of the most important household incomes and information on food security.

#### Generating indicators of household wealth

Three approaches were used to generate a measure of relative household wealth. The first used data only from the participatory wealth ranking; the second used data from the household survey, but with their selection and weighting informed by PWR; the third used only data from the survey, employing principal components analysis (PCA) to determine the weights.

##### Method 1: an index of household wealth from PWR

Details of the scoring method used are provided in detail elsewhere [24]. Briefly, within each of the three ranking processes, piles of households were assigned a score such that the wealthiest pile received a score of 100 and the poorest pile a score of 0. Scores for the remaining piles were calculated as Score for pile n = 100*((N-n)/(N-1)), where n was the pile number and N was the total number of ranking piles.

Coded *pile statements *made in relation to the piles generated were assigned the numeric score allocated to the pile. An average *pile statement *score was calculated as the mean of the pile scores to which that statement was associated, covering the full PWR process in all eight villages (Table 1). A wealth index was calculated for each household as the mean of the *pile statement *scores of all the pile statements made in relation to the piles into which each household was ranked.

**Table 1:** Pile statement scores and frequency of statements made during participatory wealth ranking in rural South Africa, in descending order of pile statement score

**Pile statements**		**Theme**	**Statement**	**General statements**		
				
				**No. of times said**		
			
**Pile statement score**	**No. of times said**			**Very poor**	**Poor but a bit better off**	**Doing OK**
0.0	22	Family and household	Orphanhood/no parents	24		
0.4	39	Food	Beg for food	33		
1.1	85	Begging	Begging	49		
3.1	134	Food	No food	137		
3.2	41	Housing	Not got shelter	33		
3.7	58	Employment	No one is working	34		
5.2	101	Schooling	Doesn't go to school	39		
5.6	73	Clothing	No clothes/do not have clothes	73		
5.7	199	Employment	Not got job(s)/unemployed	113		
5.8	22	Food	Sleep without food	17		
6.4	82	Money	Don't have/earn money/income	49		
8.3	100	Schooling	Unable to/can't afford to go to school	66		
9.0	67	Housing	Not got housing	65		
9.6	37	Schooling	Cannot afford/does not pay school fee	18		
11.9	23	Clothing	Tattered/torn/poor clothes	20		
14.4	76	Housing	Shacks	18		
15.0	51	Housing	No proper housing/shelter	18		
22.7	64	Housing	Bad/poor housing	19		
24.0	175	Employment	Farms		80	
28.4	145	Self employment	Selling fruits and vegetables		39	
28.5	71	Food	Mealy meal only		37	
28.7	99	Employment	Domestic work		45	
29.9	60	Pensions	Pension and many responsibilities		25	
34.7	26	Food	At least have food		19	
35.1	28	Food	Little food		33	
35.7	47	Self employment	Self employed		17	
38.2	55	Clothing	Second-hand clothes		21	
39.4	70	Money	Little money/income/earn less		29	
39.8	44	Housing	Small/little housing		26	
40.4	25	Schooling	Attains Matric/std 10/grade 12		17	
44.4	70	Pensions	Receiving pension		16	
61.4	79	Schooling	Able to/affords to go to school		29	
65.4	28	Employment	Got jobs/employed		24	18
71.0	32	Clothing	Good clothes			58
78.6	26	Clothing	Children have good clothes			30
80.8	134	Self employment	Taxis			41
83.1	104	Cars	Have/drive cars			50
84.7	101	Employment	Government			26
84.8	162	Schooling	Attains university/tertiary			52
86.4	97	Employment	Both husband and wife employed			18
87.9	163	Housing	Big house			96
90.1	123	Schooling	Private/expensive			76
90.4	73	Housing	Beautiful/attractive housing			42
93.8	65	Self employment	Has a business			47
95.5	74	Self employment	Shop owners			32
95.6	142	Cars	Have/drive expensive/flashy cars			102
95.7	47	Housing	Tiled housing			21

##### Method 2 : an index of household wealth from household survey data informed by PWR

Survey data were used to generate an indicator of household wealth, using PWR to inform which factors to use and how to weight the data. Where data were available on aspects of household wealth relating to each of the 10 commonest themes identified in PWR, this was used to inform the calculation of the index of household wealth (Table 2). Broadly, where relevant PWR *pile statements *identified "very poor" households, the most related survey item was given a score of -2, and where relevant statements identified households "doing OK" the associated survey item was scored 2. A sliding scale for intermediate situations was developed where this was possible. For school attendance, scoring was stratified on the basis of age. On the basis of this scoring system, each household could receive a maximum score of 9 (wealthiest) and a minimum score of -10 (poorest).

**Table 2:** Statement scores for poverty statements from PWR and scores for indicators collected in the survey data to create household index of wealth

**Theme(s)**	**Relevant statements (score)**	**Relevant data from survey**	**Score applied to survey data**
Employment, Self employment, Pensions, Money	Shop owners (95.5)	More than one household member has a salaried job	2
	Has a business (93.8)	*Either *one household member has a salaried job, *or *three or more have a pension or other work	1
	Both husband and wife employed (86.4)	No household members have a salaried job, but two has a pension or other work	0
	Government (84.7)	No household members have a salaried job, but one has a pension or other work	-1
	Taxis (80.8)	No household members have a salaried job, pension or other work	-2
	Got job/employed (65.4)		
	Receiving pension (44.4)		
	Self employed (35.7)		
	Pension and many responsibilities (29.9)		
	Domestic work (28.7)		
	Selling fruits and vegetables (28.4)		
	Farms (24.0)		
	Don't have/earn money/income (6.4)		
	Not got job(s)/unemployed (5.7)		
	No one is working (3.7)		

Schooling	Private/expensive (90.1)	If there are 20–25 year olds, if any attending or already achieved technikon or university	2
	Attains university/tertiary (84.8)	If there are 14–19 year olds and all are in school	1
	Able to/affords to go to school (61.4)	If there are 7–13 year olds and all are in school **OR **If there are 14–19 year olds and any are not attending school **OR **If no 7–25 year olds in household	0
	Attains matric (40.4)	If there are 7–13 year olds and any are not attending school	-2
	Can not afford/doesn't pay school fees (9.6)		
	Unable to/can't afford to go to school (8.3)		
	Doesn't go to school (5.2)		
		
		**Overall score; if there were young people from more than one age group in the household the average of the three scores was used**	

Housing	Tiled housing (95.7)	Face bricks	2
	Beautiful/attractive housing (90.4)	Block bricks with cement covering	1
	Big house (87.9)	Mud bricks, or block bricks without cement covering	0
	Small/little housing (39.8)	Tin or mud and sticks	-2
	Bad/poor housing (22.7)		
	No proper housing/shelter (15.0)		
	Shacks (14.4)		
	Mud housing (13.3)		
	Not got housing (9.0)		
	Not got shelter (3.2)		

Food, begging	Little food (35.1)	Food insecurity score 2–3	1
	At least have food (34.7)	Food insecurity score 4	0
	Mealy meal only (28.5)	Food insecurity score 5–6	-1
	Sleep without food (5.8)	Food insecurity score 7–8	-2
	No food (3.1)		
	Begging (1.1)		
	Beg for food (0.4)		
		
		Sum of two questions about the frequency of poor food security during the last month* pre-scored as Never (1), Once only (2), A few times (3), Often (4).	

Cars	Have/drive expensive/flashy cars (95.6) Have/drive cars (83.1)	Own any cars	2

Family and Household	Widows 1.8, n = 15^ Orphanhood/no parents (0.0)	Female Headed Household AND/OR Household consists only of children/young people	-2

* The two questions were During the last month how often a) have most of the family had a meal that consisted of pap alone, bread alone or worse, and b) have you or any of your own children gone without food or had a reduced amount to eat for a single day because of a shortage of food?

^ This statements is not listed in Table 1 because it was made less than 15 times in one stage, but was the second most common single statement about family and household made during the PWR process

##### Method 3 : an index of household wealth from household survey data with weightings assigned by PCA

Fourteen variables capturing salient aspects of household wealth, decided upon *a priori *following literature review and piloting in the local area, were made available for entry into the PCA. Items included were not limited to durable assets [5] (Table 3). Asset values were derived from the survey data by multiplying the number of owned assets that were new (less than 2 years), relatively new (2–6 years), or old (>6 years) by estimations of the value of those assets, which came from a small sub-study. Other variables were drawn from the questionnaire. Non-continuous variables were coded even-spaced ordinally.

**Table 3:** Distribution of indicators of household wealth from survey data

**Indicator**	**Variables considered for PCA** **Mean (SD), Range**	**Groupings**	**N**	**%**
		
**N**				
**Estimated value of selected non-livestock assets per person ^a ^(Quintiles)**	1548 (3211), 0–76664	0 ZAR	415	28.2
		1–131 ZAR	173	11.8
		132–348.5 ZAR	293	19.9
		350–1100 ZAR	295	20.1
		> 1100 ZAR	295	20.1
**Estimated value of selected livestock assets per person ^a ^(Quintiles)**	873 (1809), 0–28160	0 ZAR	468	31.6
		1–220 ZAR	120	8.1
		220–1115 ZAR	300	20.3
		1115–2440 ZAR	296	20.0
		> 2440 ZAR	297	20.1
**Land tenure^b^**	0.3 (0.5), 0 (no) – 1(yes)	No	1070	72.3
		Yes	410	27.7
**Quality of house wall material^a^**	3.9 (1.5), 0 (poorest) – 6 (best)	Poor	807	54.5
		Good	675	45.5
**Quality of toilet facility**	1.8 (0.4), 1 (no facility) – 3 (modern)	No facility	272	18.4
		Basic	1195	80.7
		Modern	14	1.0
**Household Electricity^b^**	0.7 (0.5), 0 (no) – 1(yes)	No	468	31.6
		Yes	1012	68.4
**Accessibility of water supply^b^**	1.7 (0.5), 1(low) – 3 (good)	Low	489	33.1
		Medium	929	62.9
		Good	60	4.1
**Density of household living conditions ^a^**	0.9 (0.8), 0.1–8 rooms per person	<= 1 rm per person	1127	76.2
		>1 rm per person	352	23.8
**Proportion of household members receiving a regular income^a^**	0.2 (0.2), 0–1	0	292	19.7
		Less than 25%	560	37.8
		25–49%	408	27.5
		50% or more	222	15.0
**Educational level of household head^a^**	3.0 (1.7), 1 (illiterate)-8 (university)	No schooling	562	38.0
		Attended primary	546	36.7
		Attended secondary or more	372	25.1
**Percentage of household members working age adults^b^**	0.6 (0.2), 0–1	50% or less	558	37.9
		>50%	915	62.1
**Gender of household head**	0.6 (0.5), 0 (female) – 1(male)	Female	587	39.6
		Male	894	60.4
**Second most important household income^b^**	0.6 (0.5), 0 (Non-financial)-1(financial)	Non-Financial	561	37.9
		Financial	921	62.1
**Regularity of household having a meal consisting of mielie meal alone, bread alone or worse**	2.3 (1.2), 1 (Often)-4 (Never)	Often	525	35.5
		A few times	413	27.9
		Once only	136	9.2
		Never	407	27.5
**Car ownership^c^**	-	No	1200	81.0
	-	Yes	281	19.0
**Schooling (7–13 yrs)^c^**	-	Any not attending	35	3.5
	-	All attending	958	96.5
**Schooling(14–19 yrs)^c^**	-	Any not attending	177	19.2
	-	All attending	747	80.8
**Schooling (20–25 yrs)^c^**	-	All not achieved college or techikon	692	90.6
	-	Any achieved college or technikon	72	9.4

^a ^denotes variables grouped for presentation in table, but where an ordered or continuous variable was used in the PCA analysis.

^b ^denotes variables considered for inclusion in the principal components analysis but not included in the final analysis

^c ^denotes variables not considered for inclusion in the principal components analysis

Non-livestock assets comprised cars, televisions, hi-fis, fridges, bicycles, cellphones. Livestock assets were cows, goats, chickens.

Low accessibility of water supply was defined as those collecting rain or stream water, medium level access was through a borehole or tap in the village, while those with high quality access were those with a tap in the plot of the dwelling.

ZAR = South African Rand

Two factors not associated in the expected direction with the value of selected non-livestock assets per person (screening variable) in a χ^2^-test (p < 0.05) were not included in the PCA (percentage of household members of working adult age and land tenure). The remaining factors were included. PCA transforms a set of correlated variables into a set of uncorrelated 'components'. When variables hold information about some underlying concept, PCA can produce the best single composite variable among all possible linear functions of the original variables [10]. The component explaining the greatest proportion of the total variance is called the first principal component. This weights the data in proportion to how well each variable is correlated with the others and was used as the indicator of household wealth.

A number of analyses were run. Factors with component loadings less than 0.2 on the first principal component were excluded (household electricity supply, quality of water supply and the nature of the second most important ranked household income). Nine factors were included in the final analysis in which the first principal component explained 22.7% of the variance of the factors in the model. The greatest weight was given to the density of household living conditions (scoring coefficient = 0.44), with the value of non-livestock assets (0.42) and the food security indicator (0.39) also being important. The lowest weighting was given to the proportion of individuals receiving an income (0.23). A wealth index was calculated for households where data were available on all variables. This component was normally distributed and had a mean of 0 and a standard deviation of 1.

#### Statistical analysis of consistency and agreement

For the PWR method only, the intra-cluster correlation coefficient, a measure of internal consistency, was first calculated from a random-effects ANOVA to describe the level of agreement in rankings of wealth between each of the three rankings made for each household [25].

Secondly, the association of each index with the individual survey indicators was estimated. Households were divided into wealth-rank tertiles on the basis of each of the methods. The association between these tertiles of wealth and each specific indicator of wealth from the survey was assessed using a χ^2^-test.

Finally, the three techniques were compared in their ranking of household wealth. The agreement of each technique placing households into wealth tertiles was estimated with a kappa coefficient. Spearman rank correlation coefficients were also calculated. While correlation coefficients measure the level of predictability of one variable on the basis of another, they do not directly assess agreement; thus a correlation coefficient of 1 will be measured if all values of one variable are twice that of another, though these clearly do not agree.

## Results

The wealth ranking process identified 9824 dwellings in 79 village sections in the eight villages, and wealth ranking data were available for 9671 of these (98.4%). Some 3556 *general statements *were coded describing the general properties of households seen as "very poor" (1240), "poor, but a bit better off" (1097) or "doing OK" (1216). A further 8856 *pile statements *were coded, describing the characteristics of the households included in each of the piles assembled by the wealth ranking process. Some 47 statements were made more than 15 times in both stages of the process (Table 1), with all but one of the statements ("Got jobs/employed") being mentioned exclusively in relation to a single wealth category. Successful interviews were completed with 1482/1640 (90.4%) households.

### Distribution and determinants of wealth

Households judged "very poor" by PWR participants were struggling to feed themselves and to clothe or educate their children, with little access to jobs or housing (Table 1). Households deemed "poor, but a bit better off" had access to low paid jobs and exhibited a basic ability to meet food and educational needs. Finally, households that were "doing OK" had access to good food, drove cars and had big or attractive housing. Some members were employed in high-return and/or high-stability activities.

Survey data (Table 3) suggested modern assets were widely distributed, though 28.2% of households reported owning none of the listed assets. Livestock assets were common. Dwellings were built of simple materials. Some 18.4% of households had no access to a toilet. Electricity supply was determined largely by village, with two villages remaining largely unelectrified. Water accessibility was generally low. Some 19.7% of households had no adults receiving a regular income, while many households were headed by an individual with no education (38.0%). Some 35.5% of households often had a meal consisting only of basic foodstuffs. Cars were owned by 19.0% of households. School attendance was high for young children but lower at later ages.

### Internal consistency of PWR

The single-measure intra-class correlation coefficient from a random effects two-way ANOVA on the three rankings of household wealth, over 9671 households, was 0.81 (95%CI0.79–0.82) denoting a high level of agreement.

### Association between wealth indices and different dimensions of wealth

Data on individual socioeconomic variables were significantly correlated (p < 0.01) with each of the wealth indices in most cases (Table 4). Land tenure was least strongly associated with the PCA measure (p = 0.026). Household electrification was not strongly associated with the measure of household wealth generated by either of the methods that used the survey data, although it was associated with the PWR index (p = 0.002). Water accessibility was least strongly associated with the PWR index (p = 0.028). Finally, the proportion of adults who were of productive age (14–60 years) was not strongly associated with household wealth as estimated by any of the techniques.

**Table 4:** The association between household wealth rank tertiles and survey indicators of socioeconomic status

	**Method 1 : PWR**		**Method 2 :Survey + PWR**		**Method 3 : Survey only**	
	**χ^2^**	**P**	**χ^2^**	**P**	**χ^2^**	**P**

Estimated value of selected non-livestock assets per person	114.9	<0.001	432.5	<0.001	445.2	<0.001
Estimated value of selected livestock assets per person	31.8	<0.001	54.4	<0.001	133.2	<0.001
Land tenure	11.7	0.003	13.0	0.002	7.3	0.026
Quality of house wall material	73.7	<0.001	219.5	<0.001	258.8	<0.001
Quality of toilet facility	38.5	<0.001	67.6	<0.001	275.5	<0.001
Household Electricity	12.6	0.002	3.1	0.21	6.3	0.044
Accessibility of water supply	10.9	0.028	23.6	<0.001	19.5	0.001
Density of household living conditions	18.5	<0.001	12.4	0.002	317.9	<0.001
Proportion of household members receiving a regular income	101.4	<0.001	188.2	<0.001	92.5	<0.001
Educational level of household head	28.6	<0.001	98.8	<0.001	155.0	<0.001
Percentage of household members working age adults	7.4	0.02	9.2	0.01	4.3	0.114
Gender of household head	64.3	<0.001	456.9	<0.001	210.6	<0.001
Second most important household income	17.2	<0.001	53.9	<0.001	11.5	0.003
Regularity of household having a meal consisting of mielie meal alone, bread alone or worse	46.4	<0.001	470.0	<0.001	539.4	<0.001
Car ownership	82.8	<0.001	354.8	<0.001	232.1	<0.001
School attendance score	23.4	0.009	83.6	<0.001	48.9	<0.001

N's for each association vary from 1442–1468 dependent on missing data.

### Agreement between the indices

The survey data methods were quite strongly correlated (Spearman rho = 0.69, p < 0.001, n = 1442), and there was a reasonable degree of agreement in their placing of households into wealth-rank tertiles (Kappa = 0.43).

The PWR wealth index was significantly, but weakly, correlated with both the index combining PWR and survey information (Spearman rho = 0.38, p < 0.001, n = 1443) and the PCA-based method (Spearman rho = 0.31, p < 0.001, n = 1451). The levels of agreement in placing households into wealth tertiles were low (kappa statistics of 0.20 and 0.17 respectively).

## Discussion

We constructed three indices of household wealth using data from a household survey and participatory wealth ranking. PWR and the survey identified similar dimensions of socioeconomic well-being as important. The two indices developed from survey data showed a reasonable level of agreement in ranking households into wealth tertiles. However, there was limited agreement between the survey-data based indices and the index based only on information from PWR. Methodological differences meant that it was not surprising that the methods differed in their results, though the magnitude of the differences noted was surprising.

The three approaches differed in at least two dimensions. The first dimension was whether information was provided by household members (as for both of the techniques using survey data), or by other community members (for the PWR only approach). The second dimension was whether community views were used to weight the importance of different aspects of wealth (as for the approaches that used PWR data), or whether external statistical rules were used (as in the PCA method). Nevertheless, there were striking similarities in the associations seen between the three wealth indices and each of the survey variables collected. The strongest associations between individual variables and the PWR wealth index were seen for variables associated at a significance level of p < 0.001 with both survey indices, while weaker associations also generally mapped across all three indices. The only exceptions to this were with the variables on household electrification and water supply.

Despite these similarities, the PWR index showed relatively low agreement with the survey-based measures, even when themes from the PWR were used to inform the selection and weighting of data. Two potential reasons for the lack of agreement are; firstly, each may have suffered from inaccurate data collection or weighting; secondly, the techniques may measure different things.

The survey attempted to maximise accurate reporting through collecting data on objective indicators, fieldworker training and stressing the importance of honesty to participants. Nevertheless, reporting biases may have occurred [26]. PWR partially accounts for this, since information is acquired from neighbours and is triangulated. However, households may conceal information from their neighbours. PWR may therefore best measure conspicuous consumption. PWR participants might also misreport household wealth. However, the high level of internal consistency for the household wealth ranks obtained from three separate groups of PWR participants provided some evidence against this. This finding differs from a previous report of low reliability for group-informant food-security ratings [27]. However, reasons for the low reliability reported by those authors were addressed in this study since trained facilitators worked with a homogenous group of PWR participants at all rankings and emphasised local definitions of poverty, participation and consensus. However, PWR may provide invalid results, but high levels of internal consistency, if participants, who were mostly poor women, ascribe a greater weight to certain dimensions of poverty (for example, being widowed) than would other groups in society.

Survey data included information on employment, educational status and asset-ownership of migrants, since temporary migrants are important contributors to the rural economy in South Africa [28-30]. However, no information was available on levels of income remittance. PWR participants may be poorly informed about the wealth of migrants or their levels of remittance. However, PWR participants may also have had a more nuanced understanding of the role of migrants in generating household wealth than it was possible to capture from the survey data.

Each method might have weighted the importance of different aspects of household wealth differently. PCA assigns weights to variables according to mathematical rules, while wealth ranking participants assess households in ways that are complex and non-transparent. Our approach to PCA incorporated different facets of wealth, as in previous applications, [5] and drew out the common underlying correlation between them. However, the first principal component explained only 22.7% of the total variance, suggesting that factors included were not well correlated. The index where PWR was used to inform the selection and weighting of survey data has intuitive appeal. However, it was not possible to directly map PWR statements to survey data, and the weighting system applied to the data was somewhat arbitrary. While combining data on multiple dimensions of socioeconomic well-being should provide a more stable marker than individual variables on their own, the selection of variables for inclusion in such analyses requires further study, as does the widespread practice of including ordered categorical and binary variables in PCA.

Finally, there was also room for differences in interpretation in PWR. Wealth ranking was conducted in *Sepedi*, applying a translation of the question, "What are the characteristics of a very poor household?" to start the ranking process. Many characteristics identified by PWR participants resonated with the survey data. Nevertheless, the way in which PWR participants judge household wealth was inevitably unclear. One possibility is that PWR participants may have ranked households more directly on their current level of welfare than the survey based methods.

In our comparison of three approaches to assessing household wealth, the method by which data were collected was more important than the method by which variables were selected or weighted in determining agreement between the rankings. None of the techniques was precise in defining what aspects of wealth they wished to measure, so ultimately the indices may have measured different things. Survey data on individual variables may be most appropriate when comparison is needed between different settings or time-periods. PCA is a useful tool for the reduction of multiple indicator data, yet in this application did not agree with household wealth ranking ascribed by community members. PWR allowed a measure of wealth to be generated for about 200 households in a given geographical area over a two-day period by a skilled practitioner. Although the use of this technique will require epidemiologists to attain new skills, PWR may represent a rapid, useful and internally valid tool for health researchers in situations where locally-grounded data on household wealth are required.

## Competing interests

The author(s) declare that they have no competing interests.
